# Precision Breast-Conserving Surgery With Microwave Ablation Guidance: A Pilot Single-Center, Prospective Cohort Study

**DOI:** 10.3389/fonc.2021.680091

**Published:** 2021-05-26

**Authors:** Hong Pan, Mengjia Qian, Hao Chen, Hui Wang, Muxin Yu, Kai Zhang, Siqi Wang, Jing Deng, Yi Xu, Lijun Ling, Qiang Ding, Hui Xie, Shui Wang, Wenbin Zhou

**Affiliations:** ^1^ Department of Breast Surgery, The First Affiliated Hospital with Nanjing Medical University, Nanjing, China; ^2^ Jiangsu Key Lab of Cancer Biomarkers, Prevention and Treatment, Jiangsu Collaborative Innovation Center For Cancer Personalized Medicine, School of Public Health, Nanjing Medical University, Nanjing, China; ^3^ Pancreatic Center & Department of General Surgery, The First Affiliated Hospital with Nanjing Medical University, Nanjing, China; ^4^ Pancreas Institute of Nanjing Medical University, Nanjing, China; ^5^ Department of Radiology, The First Affiliated Hospital with Nanjing Medical University, Nanjing, China; ^6^ Department of Ultrasonography, The First Affiliated Hospital with Nanjing Medical University, Nanjing, China; ^7^ Department of Pathology, The First Affiliated Hospital with Nanjing Medical University, Nanjing, China

**Keywords:** breast cancer, breast-conserving surgery, microwave ablation, sentinel lymph node biopsy, margin

## Abstract

**Introduction:**

Negative margins in breast-conserving surgery (BCS) are essential for preventing recurrence. The aim of this study was to determine the use of preoperative microwave ablation (MWA) in the guidance of BCS for early-stage breast cancer and access whether MWA could influence the rates of positive resection margins.

**Methods:**

From 2016 to 2018, 22 women with T1/T2 invasive breast cancer were enrolled for MWA prospectively in the guidance of BCS. US-guided MWA was performed under local anesthesia, followed by BCS and sentinel lymph node biopsy (SLNB) one week after ablation. Women who underwent palpation-guided BCS directly were included as control, and propensity score matching analysis was applied.

**Results:**

MWA was performed in 22 patients. Of the 21 MWA cases with effect information, the mean tumor size in US was 20.9 ± 6.2 mm (6-37 mm). Compared with control group (BCS directly), a lower rate of positive/close margins was observed in MWA guidance group (*P* = 0.018), and MWA caused a higher rate of accurate surgery (the largest margin ≤ 3 cm and the smallest margin ≥ 1mm, *P* = 0.042). Of these 21 patients treated with MWA, 18 were candidates for SLNB. And sentinel lymph nodes were successfully identified in all cases, and no recurrence was found with a mean follow-up of 23 months.

**Conclusion:**

For patients with T1/T2 breast cancer, the application of preoperative MWA could guide BCS accurately without impairing SLNB. Clinical trials with long-term results are required to validate MWA in the guidance for breast cancer excision.

## Introduction

Breast cancer became the most commonly diagnosed cancer worldwide, with an estimated 2.3 million new cases in 2020. Breast-conserving surgery (BCS) is accepted as a standard local therapy for early stage breast cancer (1). Advances in early diagnosis have led to increased use of BCS. Success of BCS is characterized by negative margins and a good cosmetic outcome for patient. However, BCS for breast cancer is associated with positive margins in up to 41% of cases (2) and usually require re-excision surgery to obtain negative margins (3, 4). A positive margin is defined as “ink on tumor” (any invasive cancer or ductal carcinoma *in situ* cells on ink), and a negative margin is defined as “no ink on tumor” (5). Besides, a close margin was defined as tumor within 1 mm of the inked margin (6). Most surgeons in our country would re-excise a close margin in patients intraoperatively (7). However, re-excision surgeries after the first surgery are a source of physical burden, financial burden, anxiety, and worse cosmesis for patients (8). Thus, one of the primary goals of BCS is to obtain negative resection margins.

Currently, there is no established global standard for real-time and fast intraoperative margin management in BCS. Intraoperative pathologic methods, such as frozen section analysis and imprint cytology, are often used to access margin status. However, these pathologic procedures are complexity and time-consuming, which inhibit their broader applicability worldwide (9–11). Most surgeons choose to palpate breast lesions manually, feeling for the boundaries of the typically stiff lesion. However, a large proportion of breast lesions are considered “impalpable”, that is too small or soft to detect through touch, which may attribute to inaccurate resection and obtain positive or close margins.

Minimally invasive thermal therapies (12–21), such as cryoablation, radiofrequency ablation, laser ablation, microwave ablation (MWA), have been attempted for the treatment of small breast cancer, showing high rates of tumor ablation and low rates of complication. Numerous studies of thermal ablation of breast cancer have been reported, and most are feasibility studies about ablation of small tumors (≤ 2cm) followed by immediately resection (16, 18–20). Long-term outcomes have seldom been reported (22, 23) and these thermal therapies have not been used in clinic instead of surgeries for breast cancer nowadays. However, we have to pay attention to that thermal ablation can cause a high temperature within the targeted lesion. Compared with other minimally invasive technologies, MWA shows higher temperatures, larger ablation volumes and shorter ablation times (17, 24). Furthermore, MWA is not influenced by the content of the tissue (25). The tumors one week after MWA are stiffer than before, and the ablated tumors become more palpable than before (26). We inferred that the MWA-treated breast tumor would be resected more accurately than the primary tumor.

Besides, sentinel lymph node biopsy (SLNB) are standard surgical techniques for early stage breast cancer with clinically negative lymph nodes (27, 28). Theoretically, the sentinel lymphatic channels from the subareolar plexus and/or the tumor to the axilla may be obstructed by thermal ablation of breast cancer (29, 30). It is not clear whether thermal ablation will impair the ability to perform SLNB.

The aims of this pilot study were to determine whether preoperative MWA could guide BCS for patients with clinical T1/T2 breast cancer and improve the accuracy of delayed BCS by analyzing its impact on surgical resection margins, and to determine whether MWA could affect the success rate of following SLNB.

## Materials and Methods

### Study Design

This study was performed in one group of our department from 2016 to 2018, and treatment for all breast cancer patients were discussed by the multi-disciplinary team in our center. Candidates for BCS were selected from patients diagnosed with invasive breast cancer according to the guidelines in our center. We offered two options for patients who had MWA followed by delayed BCS, or BCS directly. We would explain the advantages and disadvantages of these two types of treatments and how they were performed. The decision in this study was decided by shared decision-making between patients and operating teams. Due to limited enrollment of previous studies about MWA, the current study was not randomized in design. The pilot study consecutively included female participants treated with MWA, followed by BCS and SLNB, prospectively. The complications were assessed by the same surgeon during the MWA, the delayed surgery. Follow-up was given to patients according to the guidelines.

This study (ChiCTR1900023959) was conducted with the approval of the institutional ethics committee of The First Affiliated Hospital with Nanjing Medical University (2010-SR-003) and complied with the Declaration of Helsinki. Informed consent was obtained from all patients.

### Inclusion and Exclusion Criteria

Inclusion criteria for preoperative MWA were women older than 18 years, with invasive breast cancer confirmed by core biopsy and clinical T1/T2 tumor suitable for BCS which is clearly visible with US. The skin and muscle should not be infiltrated by the tumor. Hormone receptor and human epidermal growth factor receptor 2 (HER2) were determined before MWA. The exclusion criteria included the following: patients with an extensive intraductal component in invasive cancer, patients who were pregnant or breast-feeding, neoadjuvant therapies, and tumors located on nipple and areola area.

### Procedures

The microwave irradiation frequency of the system (Nanjing Yigao Microwave Electric Institute, Nanjing, China) was 2450 MHz with an output power set at 40 W, and cooled-shaft microwave antenna (14G) was applied in this study. Local anesthesia was induced by using 1% lidocaine. Hydrodissection in subcutaneous space and retromammary space was applied for skin protection and avoiding pains (26). About 20ml of 0.5% lidocaine was injected into both subcutaneous space and retromammary space to make both spaces wider than before. When we had to add lidocaine because of moderate-severe pain during the procedure, the procedure was stopped and 0.5% lidocaine was injected into the narrow space, either subcutaneous space or retromammary space under the guidance of ultrasound, depended on the location of pain in patients.

MWA required 1-5 min for complete ablation guided by US. All procedures were performed by a surgeon with 10 years of experience in breast intervention, including MWA technique. About one week after MWA, BCS was performed to these patients, and SLNB was given to patients who were suitable for this technique. Intraoperatively, 2 mL of methylene blue dye was injected in the subareolar area for SLNB. The surgery was performed by one surgeon with about 20 years of experience in breast surgery. Systemic treatment was recommended according to the guidelines.

### Pathologic Evaluation

Pathologists did not perform intraoperative frozen section analysis. After BCS, the size of the breast specimen was measured, and the specimen was sliced sequentially into 5-mm sections. Then, the size of the ablation zone was measured macroscopically, and cell viability was determined by 2,3,5-Triphenyl tetrazolium chloride staining (25, 31). Hematoxylin-eosin stain was also performed. Ink on tumor was defined as positive margins. The surgical margins were measured when negative margins were found, and a close margin was defined as tumor within 1 mm of the inked margin (6). Accurate surgery was defined as the largest margin ≤3 cm and the smallest margin ≥1mm. All pathological examinations were performed by two pathologists with more than 10 years of experience in breast pathologic examination independently.

### End Points

The primary end point was the proportion of positive or close resection margins. The secondary end points were the success rate of SLNB after MWA and the effect and side effects of MWA.

### Cosmetic Outcome

Four-point scoring system was used to assess cosmetic outcome from patients who completed surgery and radiation. The system rates the breast appearance on a scale that has 4 points: 1 (excellent: appearance nearly identical to the contralateral breast), 2 (good), 3 (fair), and 4 (poor: major functional and aesthetic sequelae in the treated breast). Cosmetic outcome with a fair or poor score is considered to be a cosmetic “failure”.

### Patient Satisfaction

Patient satisfaction was based on a composite questionnaire that included questions on symmetry between the two breasts on different items, including nipple position, firmness of the breast, breast volume, and breast contour. The satisfaction rating defined the level of satisfaction of patients. It included a five-level satisfaction rating: very satisfied, satisfied, neutral, dissatisfied, or very dissatisfied. Generally, “very satisfied” and “satisfied” patients were defined as the satisfied group, while “neutral”, “dissatisfied” and “very dissatisfied” patients were defined as the dissatisfied group.

### Pain Assessment

The level of pain was evaluated using the numeric rating scale (NRS). Patients rated their pain on a scale that had 11 points, from 0 to 10. Zero means “no pain”, while “10” means “the worst possible pain”. The mild pain was classified as NRS < 3, and moderate-severe pain was classified as NRS ≥ 3.

### Statistical Analysis

Numerical data were reported as the mean ± standard deviation. The BCS specimen in our department was cuboid, so the volume was calculated by using the three-dimensional axis (a, b, c) with the equation *V* = a*b*c. The rate of positive/close margins of BCS directly in our hospital is about 40%. We estimated that the rate of positive/close margins in MWA group (MWA guided BCS) is less than 10%. In the present study, the proportion of included patients in the control group (BCS directly) and MWA group is 2:1. The test statistic used is the two-sided Z test with pooled variance. The significance level of the test was targeted at 0.05, and the power (1-β) was set as 0.80. The sample size was calculated as 22 cases in the MWA group. The baseline characteristics in MWA group and control group may be not well balanced, propensity score matching analysis was performed. Differences between the two groups were analyzed with the chi-square test or Fisher’s exact test for categorical variables and the Student *t* test for continuous variables. All *P*-values were two-tailed with 5% significance levels. All analyses were performed using the software STATA version 11.0 (Computer Resource Center, America).

## Results

### Baseline Characteristics

From 2016 to 2018, 370 patients with breast cancer were treated in one group of our center. Of these 370 patients, 94 patients with invasive breast cancer were candidates for BCS. Among these 94 patients, 22 underwent MWA followed by BCS one week later, and other 72 patients underwent palpation-guided BCS firstly (see **Supplementary Figure 1**). The baseline characteristics of these 22 patients are summarized in **Table 1**.

**Table 1:** Baseline characteristics of patients who received preoperative MWA.

Variables	MWA (n = 22)
**Mean age (y)**	53.7 ± 11.0
≤50	10 (45.5%)
>50	12 (54.5%)
**Mean tumor size in US**	20.9 ± 6.2
≤2 cm	11 (50.0%)
>2 cm	11 (50.0%)
**Clinical node status**	
Negative	18 (81.8%)
Positive	4 (18.2%)
**Tumor location**	
Lateral-superior	12 (54.5%)
Interior-superior	7 (31.8%)
Lateral-inferior	3 (13.6%)
**Molecular subtype**	
HR positive and HER2 negative	10 (45.5%)
HER2 positive	5 (22.7%)
Triple negative	7 (31.8%)

HR, hormone receptor; MWA, microwave ablation; US, ultrasound.

### Preoperative MWA Guide BCS

**Figure 1:**
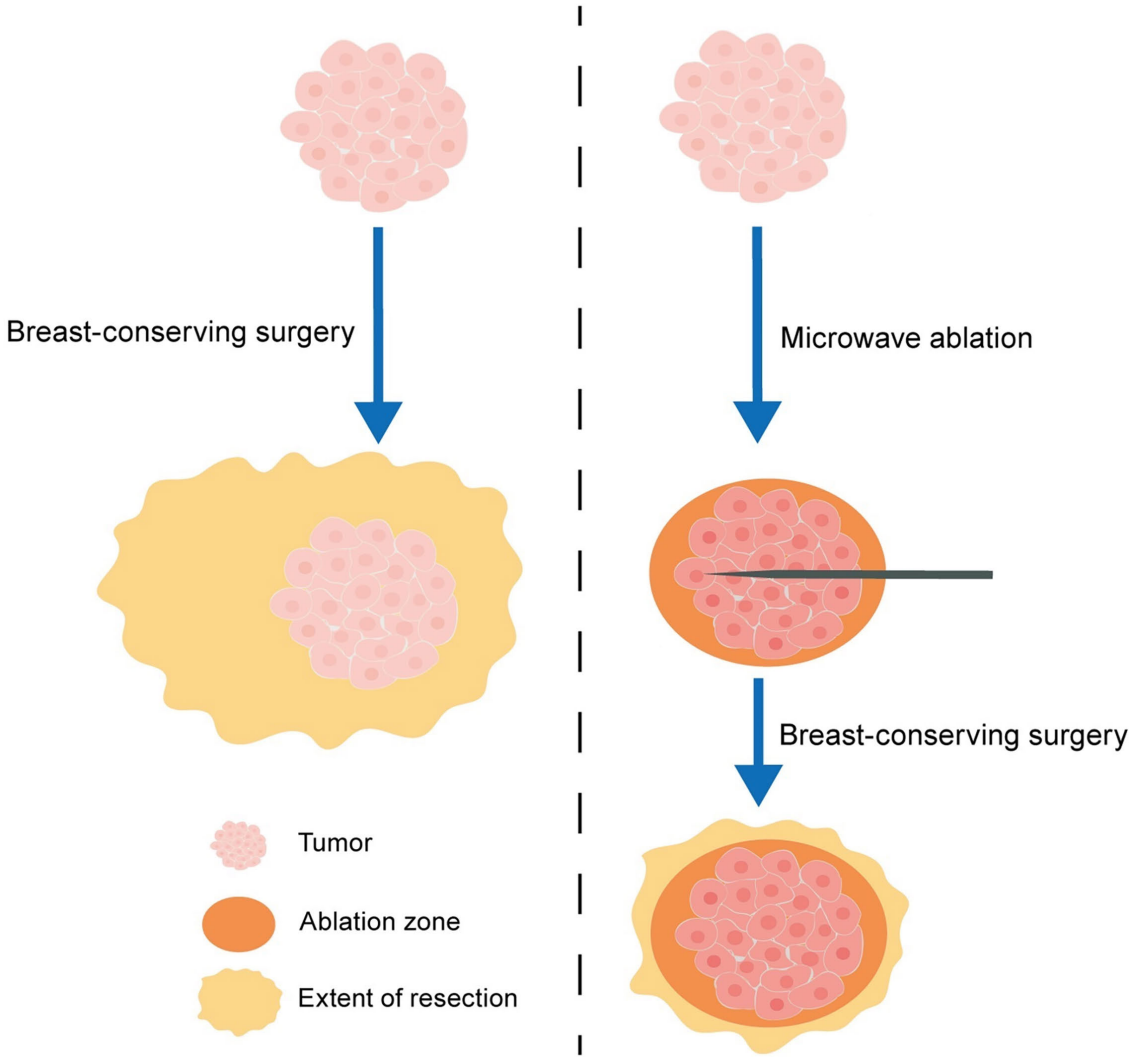
The schematic diagram of breast-conserving surgery (BCS) with microwave ablation (MWA) guidance.

To determine whether MWA can guide the following BCS, the patients who underwent BCS directly were selected as control. **Figure 1** shows the schematic diagram of BCS with MWA guidance. The baseline characteristics of these 94 patients are summarized in **Supplementary Table 1**. Because the baseline characteristics in the two groups were not well balanced, propensity score matching analysis was performed (**Table 2**). Baseline characteristics were well balanced in MWA group (n = 21) and control group (n = 42).

**Table 2:** Characteristics of propensity score-matched patients in MWA group and control group.

Variables	MWA (n = 21)	Control (n = 42)	*P* value
**Mean age (y)**	54.19 (38-72)	52.95 (32-73)	0.6559
≤50	9 (42.9%)	17 (40.5%)	0.856
>50	12 (57.1%)	25 (59.5%)	
**Mean tumor size in US**	20.9 (6-37)	20.5 (8-42)	0.8685
≤2 cm	11 (52.4%)	27 (64.3%)	0.363
>2 cm	10 (47.6%)	15 (35.7%)	
**Tumor location**			
Lateral-superior	11 (52.4%)	23 (54.8%)	0.781*
Interior-superior	7 (33.3%)	10 (23.8%)	
Lateral-inferior	3 (14.3%)	7 (16.7%)	
Interior-inferior	0 (0%)	2 (4.8%)	
**Molecular subtype**			
HR positive and HER2 negative	10 (47.6%)	22 (52.4%)	0.882*
HER2 positive	4 (19.0%)	9 (21.4%)	
Triple negative	7 (33.3%)	11 (26.2%)	

*Fisher’s exact test.

HR, hormone receptor; MWA, microwave ablation; US, ultrasound.

Regardless of the viability of the tumor after MWA, positive margins were found in one of 21 cases (4.8%) in MWA group and 6 of 42 cases (14.3%) in control group (**Table 3**). Importantly, a lower rate of positive/close margins in MWA group was observed in comparison to that in control group (2/21 vs 16/42, *P* = 0.018, **Table 3**). The accurate margins were measured in 20 MWA cases and 40 control cases. There was a larger mean resected volume in MWA group than that in control group (152.0 mL vs 96.0 mL, *P* = 0.014, **Table 3**). Additionally, 2 of 20 MWA cases (10%) and 7 of 40 control cases (17.5%) had a largest margin >3cm. In control group, 4 cases had both a positive/close margin and a largest margin >3cm, and no similar situation was observed in MWA group. In all, MWA caused a higher rate of accurate surgery than control (81.0% vs 54.8%, *P* = 0.042).

**Table 3:** Pathologic outcomes of propensity score-matched patients in MWA group and control group.

Variables	MWA (n = 21)	Control (n = 42)	*P* value
**Positive margin**	1 (4.8%)	6 (14.3%)	0.408*
**Positive/close margin**	2 (9.5%)	16 (38.1%)	0.018
**Mean specimen volume (mL)** ^‡^	152.0 ± 102.9	96.0 ± 67.8	0.014
**Largest margin > 3cm** ^‡^	2 (10.0%)	7 (17.5%)	0.704*
**Accurate surgery**	17 (81.0%)	23 (54.8%)	0.042
**Tumor size at pathology**			
≤2 cm	6	22	0.073
>2 cm	15	20	
**Axillary status**			
Negative	14	32	0.422
Positive	7	10	

*Fisher’s exact test. ^‡^available in MWA (n=20) and control (n=40) group.

MWA, microwave ablation.

Accurate surgery is defined as the largest margin ≤ 3 cm and the smallest margin ≥ 1mm.

### Effect of MWA

**Figure 2:**
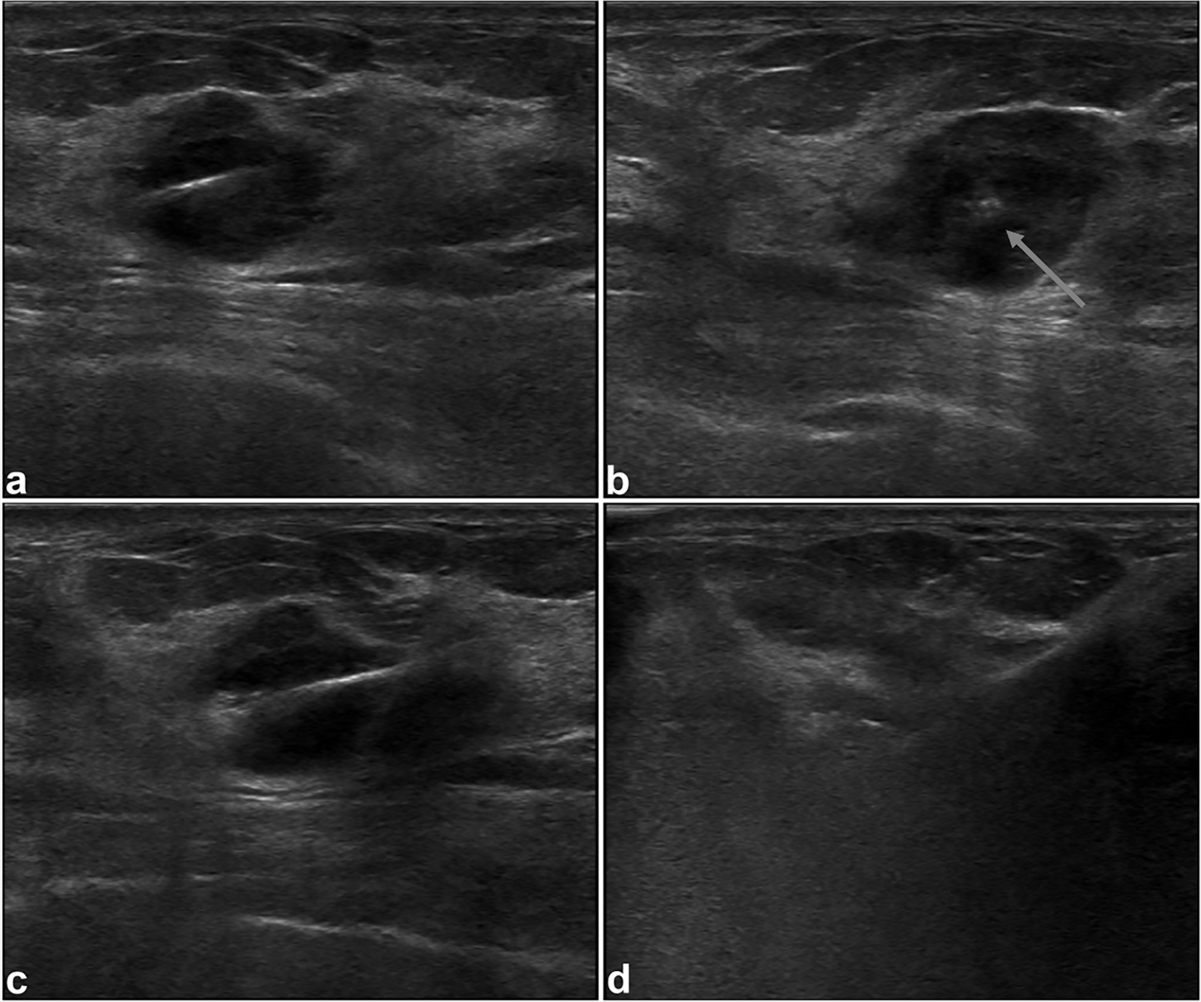
Intraoperative US images in 38-year-old woman. Longitudinal **(A)** and coronal **(B)** sonograms confirm the central placement of the antenna (arrow) within the tumor. During the procedure of MWA, sonogram shows increased echogenicity **(C)** of the tumor, and the tumor is obscured **(D)** at the end of MWA.

Of these 22 patients, one had multiple tumors, and the effects of MWA in the treatment of breast cancer were assessed in 21 patients (**Table 2**). Of these 21 patients, 10 had a single tumor with the largest diameter >2cm, and one had a single tumor >3cm. US-guided MWA were successfully performed to these 21 patients under local anesthesia (**Figure 2**), with a mean duration of 2.52 ± 0.65min (range of 1.17-4.0 min).

**Figure 3:**
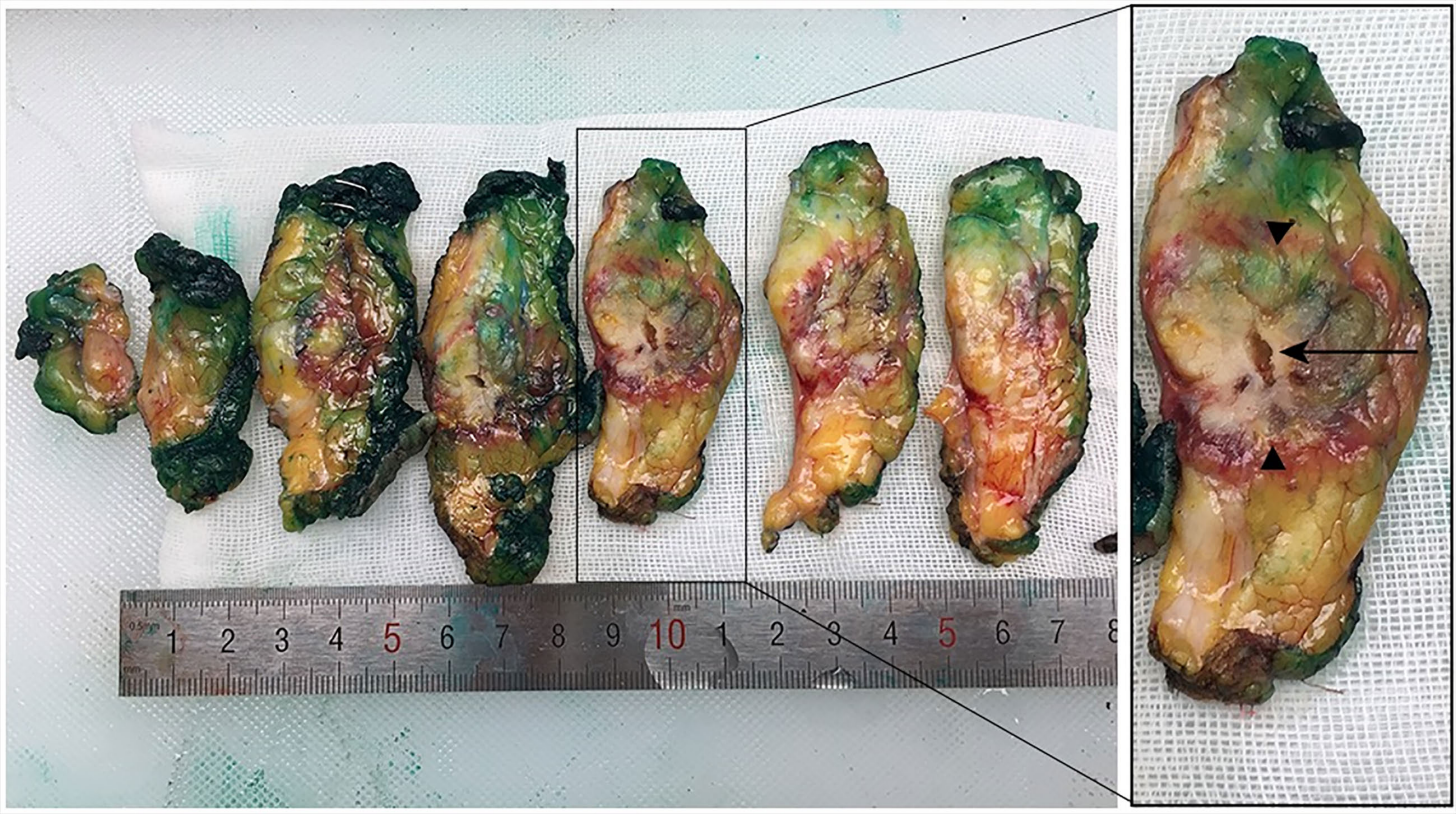
Macroscopic evaluation of excised specimen after breast-conserving surgery. The ellipsoidal ablation zone (arrowhead), surrounding by the red hyperemic area, was easily identified in all specimens, with the antenna track (arrow) in the center of the zone.

**Figure 4:**
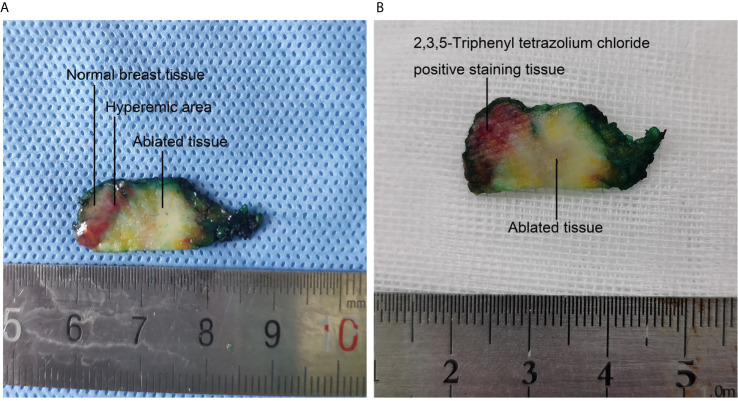
Macroscopic appearance and 2,3,5-Triphenyl tetrazolium chloride staining of one excised specimen after surgery. **(A)** The ablated tissue, hyperemic area and normal breast tissue are easily identified macroscopically. **(B)** 2,3,5-Triphenyl tetrazolium chloride staining of the specimen after surgery. The viable tissue is stained with red, and the ablated tissue is white.

One week after MWA, BCS was performed. In gross specimens (**Figure 3**), the ellipsoidal ablation zone, surrounding by the red hyperemic area, was easily identified in all cases, with the antenna track in the center of the zone. No viable cells were found in the ablation zone confirmed by 2,3,5-Triphenyl tetrazolium chloride staining (**Figure 4**). Hematoxylin-eosin stain of the ablated tissue showed definite coagulative necrosis (see **Supplementary Figure 2**). After pathological examinations, complete ablation was found in 20 of the 21 cases, and no tumor cells were found beyond the ablation zone. The extensive intraductal component, not found before MWA by X ray, US and MRI, was found in the only one case without complete ablation. Of 20 complete ablation cases, accurate sizes of the ablation zone were measured in 18 cases. MWA with a mean duration of 2.6 min can create an ablation zone of 3.14cm×2.4cm×2.02cm.

### Side Effects of MWA

In total, 54.5% (12/22) of patients had no pain, 31.8% (7/22) of patients had mild pain, and 13.6% (3/22) of patients had moderate-severe pain during MWA procedure. Due to moderate-severe pain, additional local anesthesia was given to the 3 patients during the MWA procedure, and prescheduled MWA was completed. Slight skin burn around the incision was observed in one case due to a short distance between the incision and the tumor. There were no bleeding, infection, or other adverse effects noted in 21 patients during the operation and post operatively.

### SLNB After MWA

Of these 21 patients treated with MWA, 18 were candidates for SLNB. To determine if the ability to perform SLNB was impaired after MWA, SLNB was performed to these 18 patients. Sentinel lymph nodes (median 2, range 1-4) were successfully identified in all these 18 patients, including 11 cases with the tumor located in lateral-superior area. Of these 18 cases, 13 were sentinel nodes negative, 4 were found with macrometastasis, and one was found with isolated tumor cells. Of these 4 cases with macrometastasis in sentinel lymph nodes, axillary lymph node dissection was performed to one case. With a mean follow-up of 23 months (range, 9-45 months), no recurrence was found in these 18 cases.

### Cosmetic Outcome

Cosmetic outcome data were collected from 144 patients who completed surgery and radiation included in the study. Among patients in control group who received BCS directly, 86.1% of patients (62/72) had a good or excellent cosmetic outcome when assessed by doctors. Among patients in MWA group, 86.4% of patients (19/22) had the outcome as good or excellent. There was no significant difference between the two groups (Fisher’s exact *P* = 1.000).

### Patient Satisfaction in Different Groups

We collected a survey from patients to investigate patient satisfaction in each group. Among patients in control group who received BCS directly, the rate of patient satisfaction was 80.6% (58/72). Among patients in MWA group, the rate of patient satisfaction was 86.4% (19/22). There was no significant difference between the two groups (Fisher’s exact *P* = 0.754).

## Discussion

Minimally invasive thermal ablation has been attempted in the treatment of small breast cancer (12). Recently, most reported data focused on invasive breast cancer smaller than 2 cm, and surgery was performed immediately after ablation (16–18, 21). Interestingly, the coagulation volumes induced by MWA and radiofrequency ablation reach the maximum on day 2, and the enlarged zone is caused by conductive heating of residual thermal energy after ablation (32, 33). In our opinion, the effect of thermal ablation should be assessed two days later. We found MWA with a mean duration of 2.6 min can create an ablation zone of 3.14cm×2.4cm×2.02cm, which was larger than that in the previous feasibility study with the same output power (17). We firstly reported successful experience of MWA in the treatment of T1/T2 breast cancer under local anesthesia, with a high complete ablation rate (95%). However, Training is important for accurate MWA in the treatment of breast tumors. In the present study, all procedures were performed by a surgeon with 10 years of experience in breast intervention, including MWA technique. Based on the results of our previous study (34), at least 30 cases training is recommended for doctors without experience in MWA.

We assumed that the MWA-treated breast tumor would be resected more accurately than the primary tumor. In our country, a high rate of reoperation after BCS was not acceptable. Most surgeons in our country would re-excise a close margin intraoperatively to avoid reoperation after the first surgery. In a nationwide cross-sectional survey of 110 hospitals (7), the reoperation rate after BCS was less than 5%, which indicates that surgeons may perform more extensive resection during BCS in China. Therefore, the positive/close margin intraoperatively is very important in the clinical practice in our country. In this study, we found that less positive/close margins were observed in MWA group compared with control group, partly due to a larger resected volume. Otherwise, less cases with a largest margin > 3cm were found in MWA, although no significant difference existed. Moreover, 4 cases had both a positive/close margin and a largest margin > 3cm in control group. A higher rate of accurate surgery in MWA group does not all attribute to larger resection volumes (21).

Several techniques are currently used for intraoperative margin assessment, such as intraoperative ultrasonography, optical coherence tomography (OCT), and fluorescent probe. However, ultrasound is operator-dependent and has limited reliability for visualizing *in situ* or multifocal cancers (35). Fluorescent probe potentially enable surgeons to visualize the excised lump, but there are several barriers to clinical translation (36). OCT was reported to assess margins in BCS with relatively low accuracy, which may due to the limited ability of OCT to distinguish between tumor and surrounding normal stroma (37, 38). Different from the no accurate palpation-guided BCS (39, 40), our results suggested MWA-treated tumor can guide BCS, due to the more palpable tumor after MWA than before (26). During surgery, it is actually palpable-guided surgery, which is much easier for surgeons with short learning curve. The resected specimens were larger than the ablated zone, and almost all cases were completely ablated in this study. It may be safe and accurate for BCS after MWA with only the ablated tissues resected. Interestingly, the thermal ablation induced immune response has been reported in several solid tumors (41–43). MWA may not only guide the delayed BCS, but also induce immune response. This promising treatment strategy should be tested in the future.

As a standard therapy, SLNB has been proved to be a valid method of assessing node status for early breast cancer patients with clinically negative lymph nodes (27, 28). Sentinel lymphatic channels to the axilla may be obstructed by MWA of breast cancer, especially for tumors located in lateral-superior area (29). We found that SLNB was successfully performed in 18 patients, including 11 cases with the tumor located in lateral-superior area. In our opinion, accurate ablation without too much normal breast tissue ablated may contribute to this high success rate, and future studies with large sample sizes are needed to confirm our results.

Our study has several limitations. First, patients, who received preoperative MWA guiding BCS, were enrolled in one group of our center, and the sample size was small. Second, tolerance of most patients during the ablation was well under local anesthesia. However, 3 patients suffered from obvious pain. There is still room to improve the tolerance during the procedure. Third, the accurate false-negative rate of SLNB was not determined in this study, but no recurrence was observed during the follow-up. However, axillary node dissection cannot be performed to determine false-negative of SLNB with negative or 1-2 positive sentinel lymph nodes when BCS was given according to current guidelines.

## Conclusion

For patients with T1/T2 breast cancer, the application of preoperative MWA could guide BCS accurately, safely removed tumors with low rates of positive/close margins. Besides, preoperative MWA won’t impair the following SLNB. Randomized clinical trials with large sample size and long-term results are required to validate MWA in the guidance for breast cancer excision.

## Data Availability Statement

The original contributions presented in the study are included in the article/**Supplementary Material**. Further inquiries can be directed to the corresponding authors.

## Ethics Statement

The studies involving human participants were reviewed and approved by the institutional ethics committee of The First Affiliated Hospital with Nanjing Medical University. The patients/participants provided their written informed consent to participate in this study.

## Author Contributions

HP, MQ and HC participated in the acquisition, analysis, and interpretation of data, prepared the manuscript, and had equal contribution to the study; HW, MY, KZ, S-QW, JD, YX, LL, QD and HX participated in data acquisition and manuscript drafting; SW and WZ contributed to the conception, design, and data interpretation, as well as revised the manuscript for important intellectual content. All authors contributed to the article and approve the submitted version.

## Funding

This work was supported by the National Natural Science Foundation of China (81771953), the Six Kinds of Outstanding Talent Foundation of Jiangsu Province (WSW-014, to Wenbin Zhou), the Natural Science Foundation of Jiangsu Province (BK20180108), and A Project Funded by the Priority Academic Program Development of Jiangsu Higher Education Institutions (PAPD).

## Conflict of Interest

The authors declare that the research was conducted in the absence of any commercial or financial relationships that could be construed as a potential conflict of interest.

## Supplementary Material

Supplementary Figure 1 Flow chart of patients in the study.

Supplementary Figure 2 Photomicrographs of hematoxylin-eosin stain in 59-year-old woman from the tissue of invasive breast cancer before and 1 week after microwave ablation. **(A)** Hematoxylin-eosin stain of tissue from core biopsy before ablation shows invasive breast cancer. **(B)** The ablated tissue displays coagulative necrosis of breast cancer, including degeneration of cancer cells and stromal fibrous proliferation.
