# Supplementary figures and images for: Precision Breast-Conserving Surgery With Microwave Ablation Guidance: A Pilot Single-Center, Prospective Cohort Study
Source: Front Oncol. 2021 May 26;11:680091. doi: 10.3389/fonc.2021.680091 (PMC8187871; doi:10.3389/fonc.2021.680091)

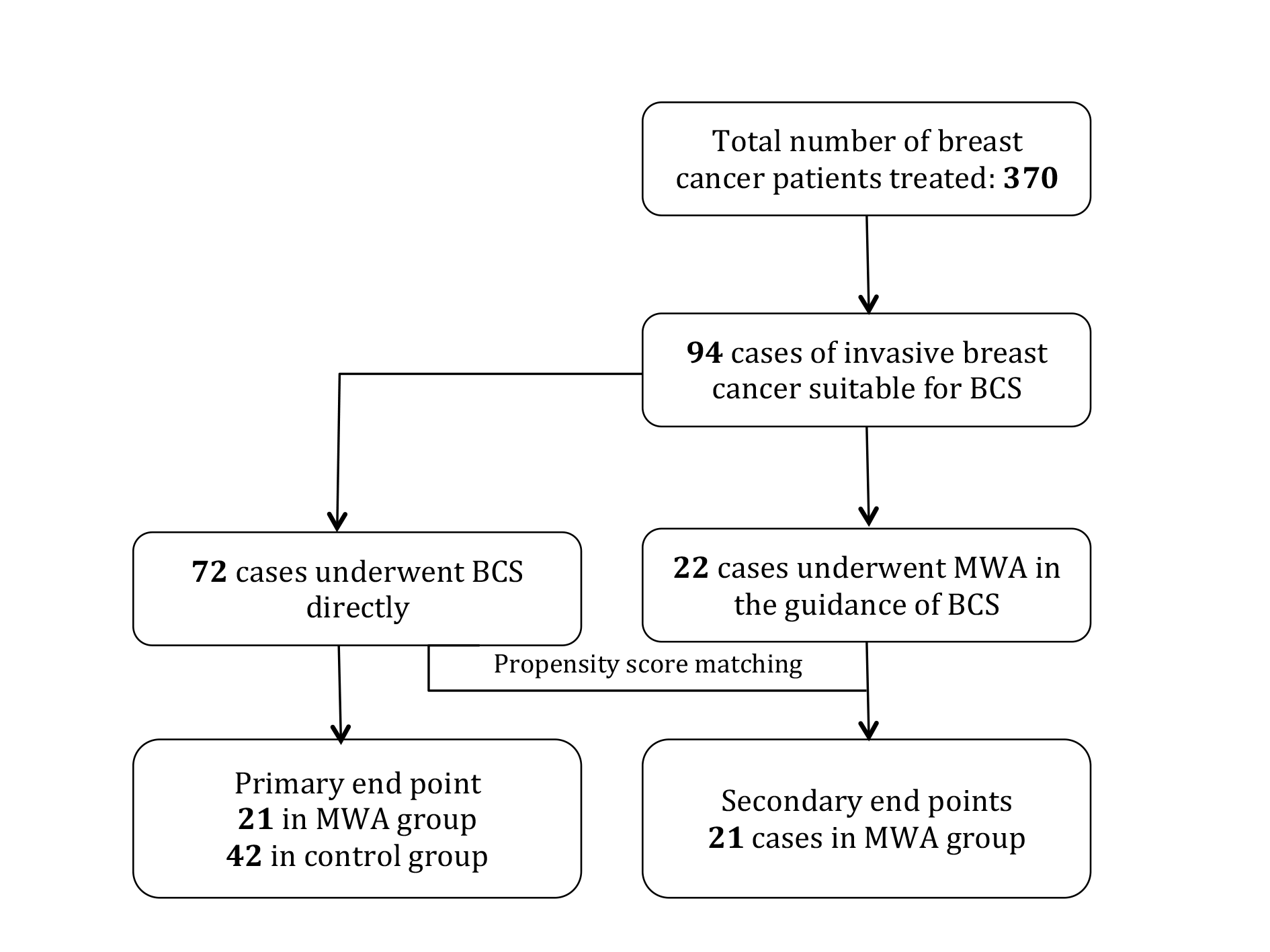

Supplement: Supplementary Figure 1 — Flow chart of patients in the study. [file Image_1.tif]

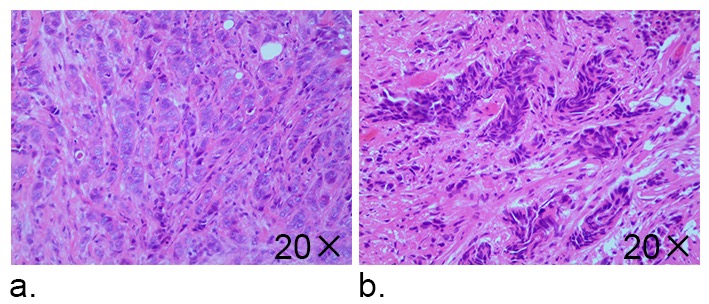

Supplement: Supplementary Figure 2 — Photomicrographs of hematoxylin-eosin stain in 59-year-old woman from the tissue of invasive breast cancer before and 1 week after microwave ablation. (A) Hematoxylin-eosin stain of tissue from core biopsy before ablation shows invasive breast cancer. (B) The ablated tissue displays coagulative necrosis of breast cancer, including degeneration of cancer cells and stromal fibrous proliferation. [file Image_2.tif]
