# Supplementary material for: Precision Breast-Conserving Surgery With Microwave Ablation Guidance: A Pilot Single-Center, Prospective Cohort Study
Source: Front Oncol. 2021 May 26;11:680091. doi: 10.3389/fonc.2021.680091 (PMC8187871; doi:10.3389/fonc.2021.680091)
Supplement: Supplementary file 3 [file Table_1.docx]

**Table S**1. Baseline characteristics of all enrolled patients in MWA and control groups

| **Variables** | **MWA (22)** | **Control (72)** | ***P* value** |
| --- | --- | --- | --- |
| **Mean age (y)** | 53.7±11.0 | 49.6±9.6 | 0.0842 |
| ≤ 50 | 10 | 24 | 0.321 |
| > 50 | 12 | 47 |  |
| NA | 0 | 1 |  |
| **Mean tumor size in US** | 20.9±6.2 | 19.6±7.0 | 0.4281 |
| ≤ 2 cm | 11 | 45 | 0.296 |
| > 2 cm | 11 | 27 |  |
| **Molecular subtype** |  |  |  |
| HR positive and HER2 negative | 10 | 47 | 0.159* |
| HER2 positive | 5 | 12 |  |
| Triple negative | 7 | 11 |  |
| NA | 0 | 2 |  |
| **Positive margin** | 1 | 14 | 0.179* |
| **Positive/close margin** | 3 | 31 | 0.012 |

*Fisher's exact test.

HR, hormone receptor; MWA, microwave ablation; US, ultrasound
